# Exposure to a 50 Hz magnetic field at 100 µT exerts no DNA damage in cardiomyocytes

**DOI:** 10.1242/bio.041293

**Published:** 2019-08-07

**Authors:** Yong Wang, Xingfa Liu, Yemao Zhang, Baoquan Wan, Jiangong Zhang, Wei He, Dong Hu, Yong Yang, Jinsheng Lai, Mengying He, Chen Chen

**Affiliations:** 1 Division of Cardiology, Department of Internal Medicine and Hubei Key Laboratory of Genetics and Molecular Mechanisms of Cardiologic Disorders, Tongji Hospital, Tongji Medical College, Huazhong University of Science and Technology, Wuhan 430030, China; 2 State Key Laboratory of Power Grid Environmental Protection, High Voltage Research Institute, China Electric Power Research Institute, Wuhan 430030, China; 3 Electric Power Research Institute of State Grid Gansu Electric Power Company, Lanzhou 730050, China

**Keywords:** Extremely low frequency magnetic fields, DNA damage, Cell cycle, Reactive oxygen species, Cardiomyocyte

## Abstract

The effects of exposure to magnetic fields (MFs) at electric frequencies (50–60 Hz) on carcinogenicity are still in debate. Whether exposure to MFs affects the heart is also a debated issue. This study aimed to determine whether exposure to extremely low frequency MFs (ELF-MFs) induced DNA damage in cardiomyocytes both *in vitro* and *in vivo*. Human ventricular cardiomyocytes were exposed to 50 Hz ELF-MF at 100 µT for 1 h continuously or 75 min intermittently. The effects of the treatments were evaluated by DNA damage, redox status changes and relative signal molecular expression. Moreover, ten male Sprague-Dawley rats were exposed to a 50 Hz MF at 100 µT for 7 days, while another 10 rats were sham exposed. The protein levels of p53 and Hsp70 in heart tissue were analyzed by western blot. The results showed that exposure to ELF-MF did not induce DNA damage, changes to cell cycle distribution or increased reactive oxygen species level. No significant differences were detected in p53 and Hsp70 expression level between the ELF-MF and sham-exposure groups both *in vitro* and *in vivo*. All these data indicate that MFs at power-frequency may not cause DNA damage in cardiomyocytes.

This article has an associated First Person interview with the first author of the paper.

## Abstract (precis)

**Summary:** The current study shows that neither continuous nor intermittent exposure to a 50 Hz magnetic field at 100 µT induces DNA damage in cardiomyocytes.

## INTRODUCTION

The relationship between extremely low frequency magnetic fields (ELF-MFs) and human health has attracted public attention for a few decades, as electric and magnetic fields (MFs) are ubiquitous in modern society. The characteristics of electric fields and MFs are determined by their sources. Since the major frequency of alternating current transmission in power systems is 50 or 60 Hz worldwide, the corresponding electromagnetic field is the same frequency; an extremely low frequency electromagnetic field (3–3000 Hz) (Jay and Goetz, 1988). In 1979, Wertheimer and Leeper reported an increased risk of childhood leukemia and nervous system tumors associated with residential ELF-MFs (Wertheimer and Leeper, 1979), which initiated the concerns about the potential carcinogenic risks of ELF-MFs. Subsequent epidemiological investigations found controversial evidence for increased risk with residential exposure to ELF-MFs (Gurney et al., 1996; Feychting and Ahlbom, 1993; Tynes and Haldorsen, 1997; Pedersen et al., 2014), and the International Agency for Research on Cancer (IARC) classified ELF-MFs as possibly carcinogenic to humans (Group 2B) in 2002 (Humans IWGotEoCRt, 2002). To date, no consensus has been reached on the carcinogenicity of ELF-MFs.

As DNA damage is correlated with cancer (Cerutti, 1985; Ames, 1989; Helleday et al., 2007), possible genotoxic effects of ELF-MFs have been widely investigated. For example, Kim et al. reported that MF exposure of 60 Hz, 7 mT for 10–60 min induced DNA double-strand breaks in human primary fibroblasts and cervical cancer cells (Kim et al., 2012). Villarini et al. conducted a study on CD1 mice exposed to 0.1, 0.2, 1 or 2 mT (50 Hz) MFs for 7 days (15 h/day) and found that DNA damage increased in all the cerebral areas of mice exposed to 1 and 2 mT MFs. Some similar studies also reported that ELF-MFs exposure induced DNA strand breaks in brain cells (Lai and Singh, 1997a,b; Mariucci et al., 2010). Additionally, Ventura et al. found that exposure of mouse embryonic stem cells to ELF-MFs triggered the expression of GATA-4 and Nkx-2.5, which could prompt these stem cells to differentiate into cardiomyocytes (Ventura et al., 2005). However, McNamee et al. did not detect any significant increase in DNA damage in rat or mouse brain cells after 2 h MF exposure (0–2 mT, 60 Hz) (McNamee et al., 2005). An *in vitro* study conducted by Su et al. investigated both neurogenic tumor cell line cells and primary cultured neurogenic cells from rats exposed to 50 Hz MFs at 2 mT for up to 24 h, and no DNA damage effects were observed (Su et al., 2017). Using different methods of exposure, Focke et al. found that intermittent (5 min on/10 min off) but not continuous exposure of human primary fibroblasts to a 50 Hz MF at 1 mT induced a slight but significant increase of DNA fragmentation (Focke et al., 2010). Nevertheless, in another study conducted by Villarini, no DNA damage was observed in human neuroblastoma cells exposed to 50 Hz MFs (0.01, 0.1 or 1 mT) for 1 h continuously or 5 h intermittently (15 min on/15 min off) (Villarini et al., 2017). Although many studies reported the genotoxicity of ELF-MFs, the conclusion was inconsistent and even controversial. This may be due to the various equipment used to generate MFs, exposure conditions, experimental protocols and biological materials adopted by different groups. In addition, ELF-MF was suspected to perturb redox-responsive intracellular signaling (Falone et al., 2018), while different cell types with specific redox status might have different cellular responses to ELF-MFs (Simko, 2007). Therefore, the effects of ELF-MFs on DNA damage still require further investigation.

In the cardiovascular system, because of the inducibility of cardiomyocytes, most studies focused on the effects of ELF-MFs on the changes in ECG or heart rate. Similarly, a consensus has not been reached since some studies observed certain effects of ELF-MFs on the heart (Maresh et al., 1988; Korpinen et al., 1993), while other studies did not (Kurokawa et al., 2003; Sait et al., 2006; Kim et al., 2013). Regardless of the debate on the effects of ELF-MFs on electric activity of the heart, no study concerning genotoxic effects of ELF-MFs on cardiomyocytes has been reported. It intrigued us to investigate the effects of 50 Hz MF exposure on DNA damage in human ventricular cardiomyocytes (AC16 cell line).

As indicated by the International Commission on Non-Ionizing Radiation Protection (ICNIRP) in 1998, the flux density limitation of general public exposure to ELF-MFs was 100 µT (ICNRP, 1998). Although the limits of general public exposure was raised to 200 µT in 2010 (International Commission on Non-Ionizing Radiation Protection, 2010), the threshold in China was still 100 µT (Administration CSSEP, 1998). Thus, here we investigated the effects of 50 Hz ELF-MF exposure on cardiomyocytes at the flux densities of 100 µT.

Different exposure timings and flux density may lead to various effects. Meanwhile, some studies reported that intermittent exposure was more likely to cause DNA damage than continuous exposure. AT478 squamous cell carcinoma cells exposed to a 50Hz ELF-MF at a flux density of 1 mT for 16 min resulted in an increase in DNA damage compared to control cells (Bułdak et al., 2012). Intermittent exposure (5 min on/10 min off, 1 mT) did not affect the mitochondrial membrane potential in human diploid fibroblasts (Pilger et al., 2004). However, ELF-MF at the intensity of 0.4 mT did not induce DNA damage after 2, 6, 12, 24 or 48 h exposure in human lens epithelial cells *in vitro* (Zhu et al., 2016). Moreover, the continuous treatment of 100 μT ELF-MF did not induce significant changes in neuroblastoma line NB69 proliferation. In contrast, intermittent exposure (5 min on/10 min off or 3 h on/3 h off) caused statistically a significant increase in the percent of neuroblastoma line NB69 in phase S of the cell cycle, followed by a significant increase in cell number (Martínez et al., 2012).

Thus, these two exposure modes were both adopted in our study; 1 h of continuous and 75 min of intermittent (15 min power field on/15 min power field off) exposure.

## RESULTS

### Exposure to ELF-MF did not increase DNA strand breaks in cardiomyocytes

**Fig. 1.**
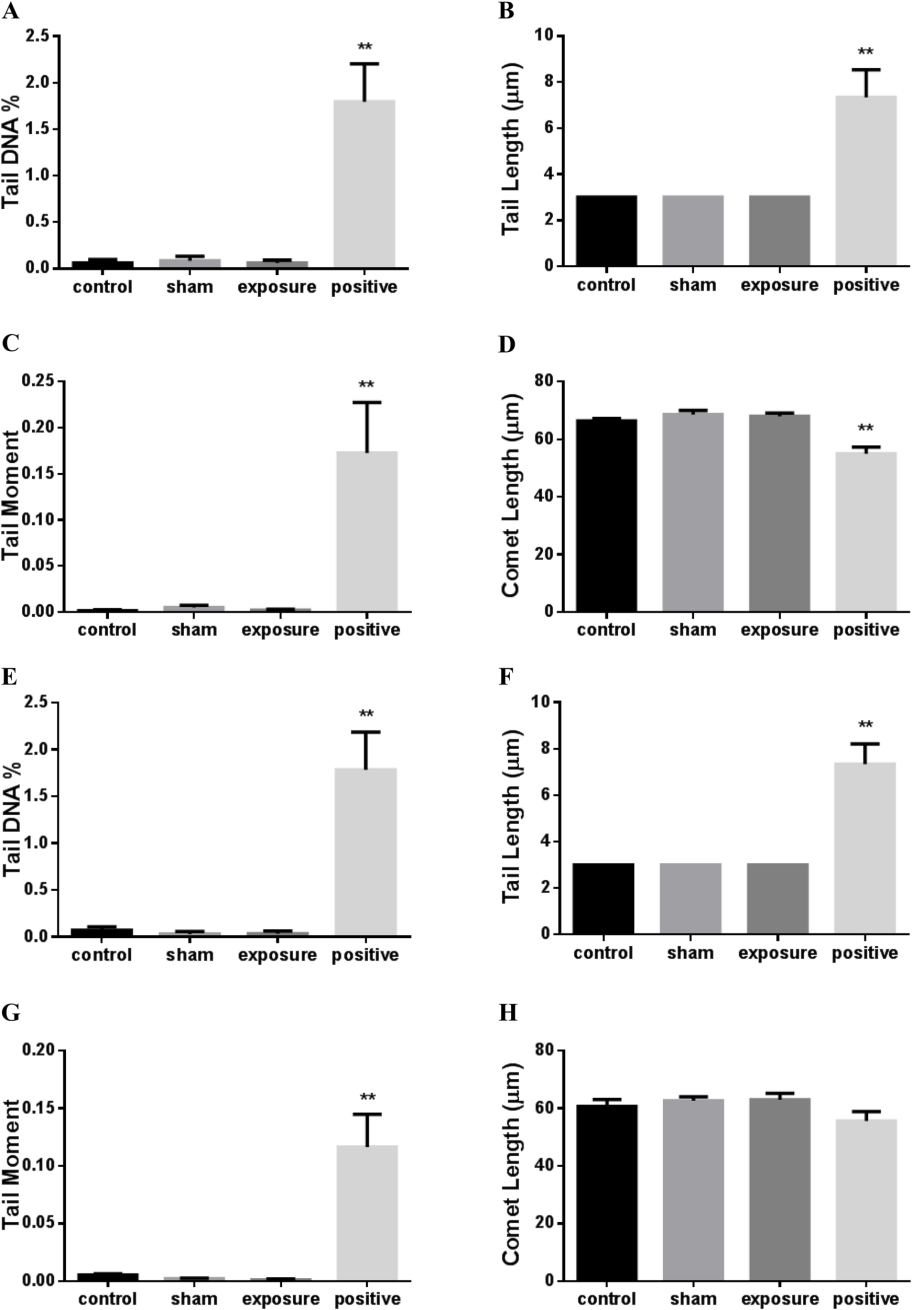
**DNA damage in AC16 cells exposed to 50 Hz MF.** Levels of DNA damage were evaluated by the alkaline comet assay following 1 h continuous (A–D) or 75 min intermittent (E–H) exposure, expressed as tail DNA %, tail length, tail moment and comet length. Negative control (control) represents unstressed cells kept under normal culture conditions. The positive control (positive) used negative control cells exposed to ice-cold 2 µM hydrogen peroxide for 5 min. Each bar shows the mean±s.e.m. of three independent experiments. A minimum of 50 cells were collected from each replica of a sample, using the mean values of the medians of the two replicas for comparison (**P<0.01 versus control). Because the CASPLab program was set to measuring the tail length with an initial value of 3 μm and a minimum increment of 1 μm. The non-normal distribution of comet made the median of several groups 3 μm.

A comet assay was used to determine DNA damage. The negative control, AC16 cell suspensions, were treated for 5 min at 4°C with 2 μM H_2_O_2_ as positive control. The parameters, including tail DNA percentage and tail length used to quantitate DNA damage, showed significant increases (Fig. 1A–H). However, no significant difference was observed in the DNA percentage, tail length, tail moment and comet length between negative control cells and sham-exposed cells (Fig. 1A–H). Moreover, compared to the sham-exposure group, no significant changes were found after continuous (Fig. 1A–D) or intermittent exposure to ELF-MF (Fig. 1E–H).

### Exposure to ELF-MF did not influence the cell cycle in cardiomyocytes

**Fig. 2.**
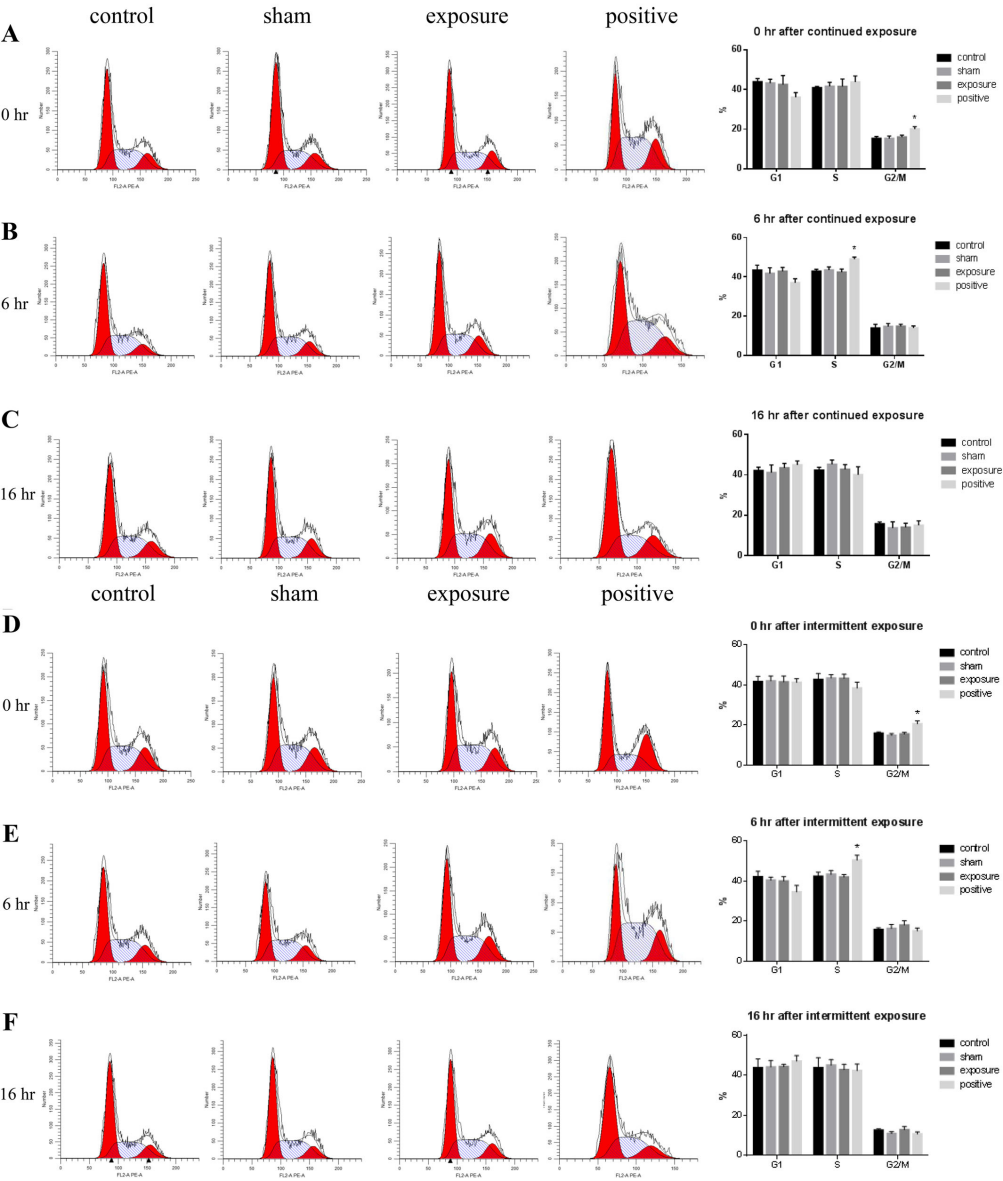
**Effects of exposure to 50 Hz MF on cell cycle distribution in AC16 cells.** The proportions of cells at each phase of the cell cycle were measured by flow cytometry at 0, 6 and 16 h after 50 Hz MF exposure for 1 h continuously (A–C) or 75 min intermittently (D–F), or after 1 μM nocodazole for 1 h as a positive control. Left, representative cell cycle profiles in negative control (control), sham exposed (sham), ELF-MF exposed (exposure) and positive control (positive) groups, from left to right. Right, histograms of percentages of cells at different phases of the cell cycle. The data represent the mean±s.e.m. from three independent experiments (*P<0.05 versus control).

To examine the genotoxic effects of ELF-MF exposure on AC16 cells, the cell cycle distributions at 0, 6 and 16 h after exposure were analyzed by flow cytometry. As expected, the 1 μM nocodazole-treated cells (positive control) showed a significant difference compared with the negative control at 0 h and 6 h (Fig. 2A,D,E). No difference was detected between the sham and the negative control at 16 h (Fig. 2C,F). More importantly, there was no significant difference in the percentage of cells at G0/G1, S or G2/M phase at each time point after continuous (Fig. 2A–C) or intermittent exposure to ELF-MF (Fig. 2D–F). These results suggest that neither continuous nor intermittent exposure to ELF-MF influences cell cycle progression.

### Exposure to ELF-MF had no effect on reactive oxygen species in cardiomyocytes

**Fig. 3.**
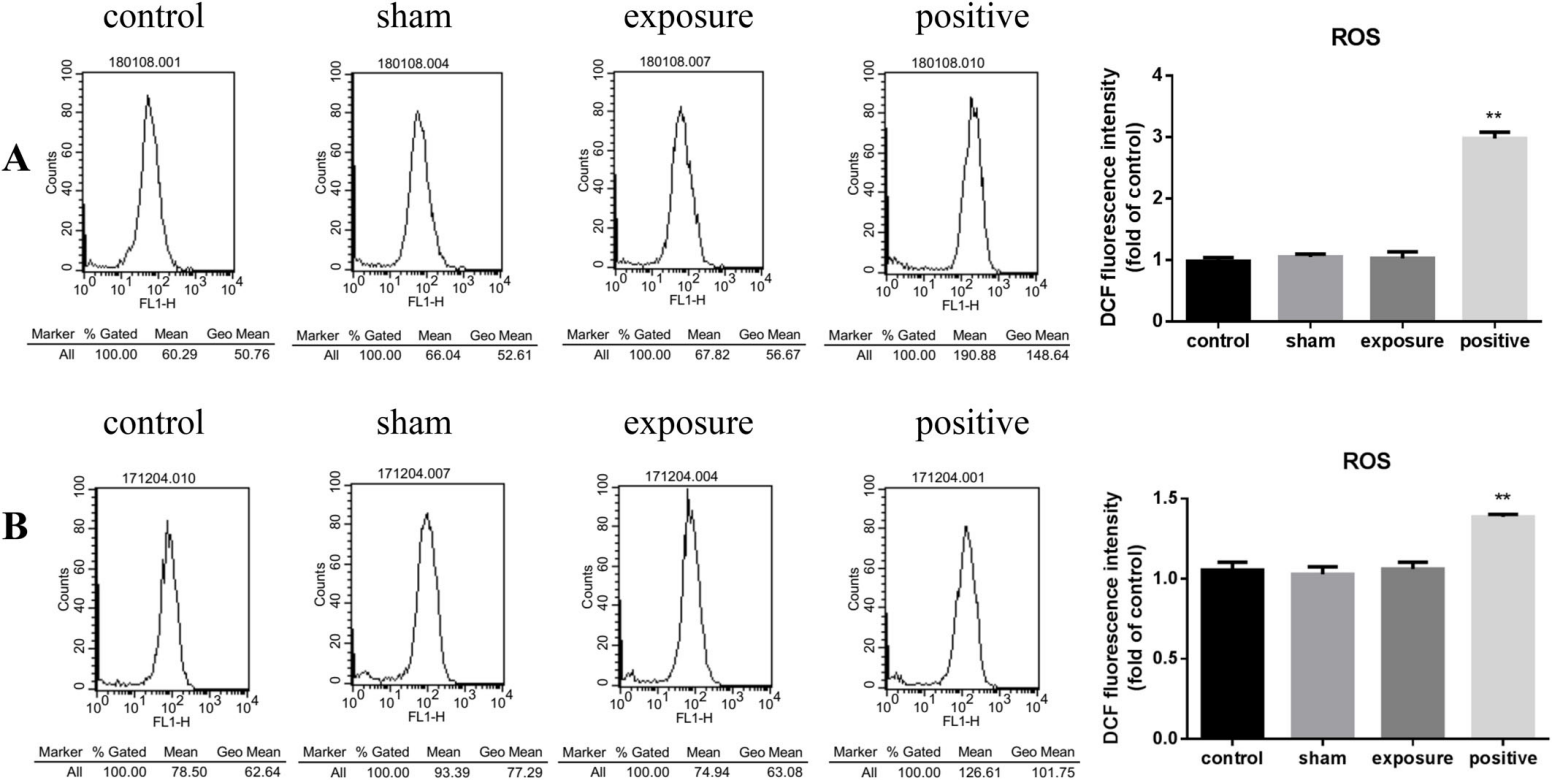
**ROS generation in AC16 cells exposed to 50 Hz MF.** Levels of intracellular ROS were measured by flow cytometry using DCFH-DA after 1 h continuous (A) or 75 min intermittent (B) exposure to 50 Hz MF. Cells treated with 200 μM H_2_O_2_ for 30 min were used as positive control. Left, representative cell DCF ﬂuorescence intensity profiles in negative control (control), sham exposed (sham), ELF-MF exposed (exposure) and positive control (positive) groups, from left to right. The vertical axis represents cell numbers, horizontal ordinate is DCF ﬂuorescence intensity. Right, histograms of geometric mean value of DCF ﬂuorescence intensity in each treatment group. The data represent the mean±s.e.m. from three independent experiments (**P<0.01 versus control).

As shown in Fig. 3, in both continuous (Fig. 3A) and intermittent (Fig. 3B) exposure modes, the DCF fluorescence intensity of the MF-exposed groups were the same as those in the negative control group. These results indicate that total intracellular reactive oxygen species (ROS) production was not changed by exposure to MF at 100 μT.

### Exposure to ELF-MF did not change the redox status in cardiomyocytes

**Fig. 4.**
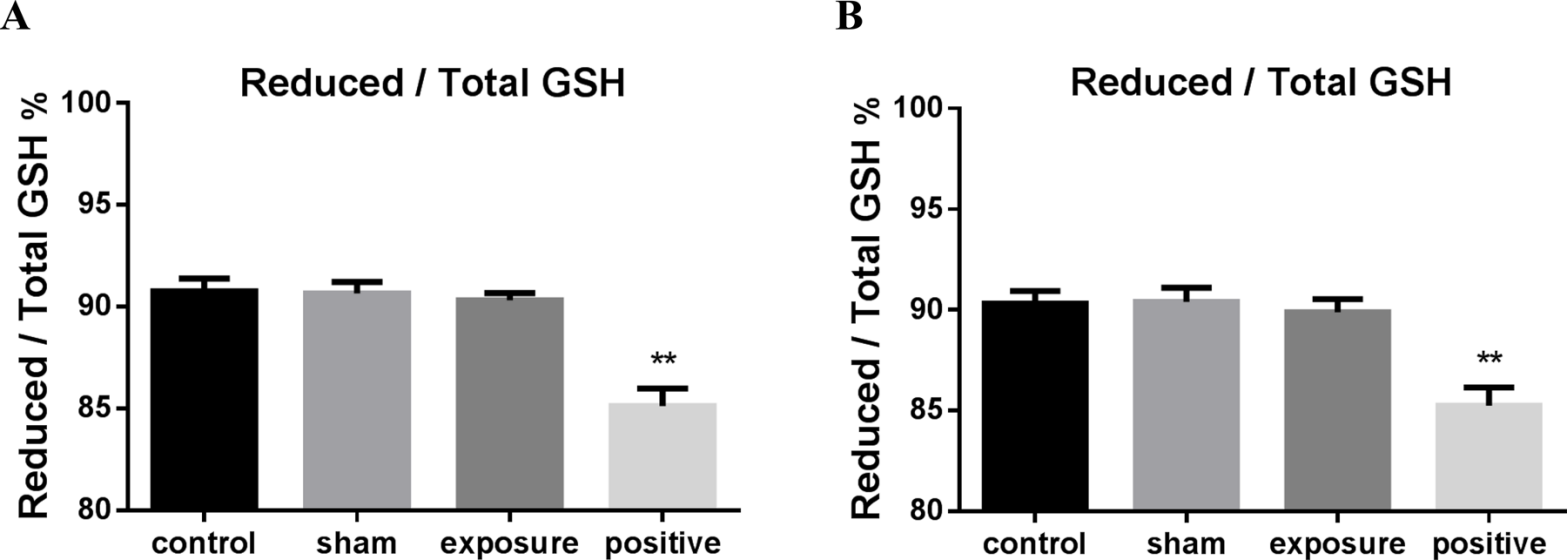
**The ratio of reduced/total GSH in AC16 cells exposed to 50 Hz MF.** Quantitative analysis of reduced/total GSH ratio in negative control (control), sham exposed (sham), ELF-MF exposed (exposure) and positive control (positive) groups after 50 Hz MF exposure for 1 h continuously (A) or 75 min intermittently (B). Cells treated with 200 μM H_2_O_2_ for 30 min were used as positive control. The data represent the mean±s.e.m. from three independent experiments (*P<0.05 versus control).

We detected intracellular GSH and GSSG to further investigate the redox status. Consistent with the results of ROS, the ratio of reduced/total GSH in the exposure groups showed no statistical significance with the control group under both continuous (Fig. 4A) and intermittent exposure conditions (Fig. 4B).

### Exposure to ELF-MF did not alter response protein expression

**Fig. 5.**
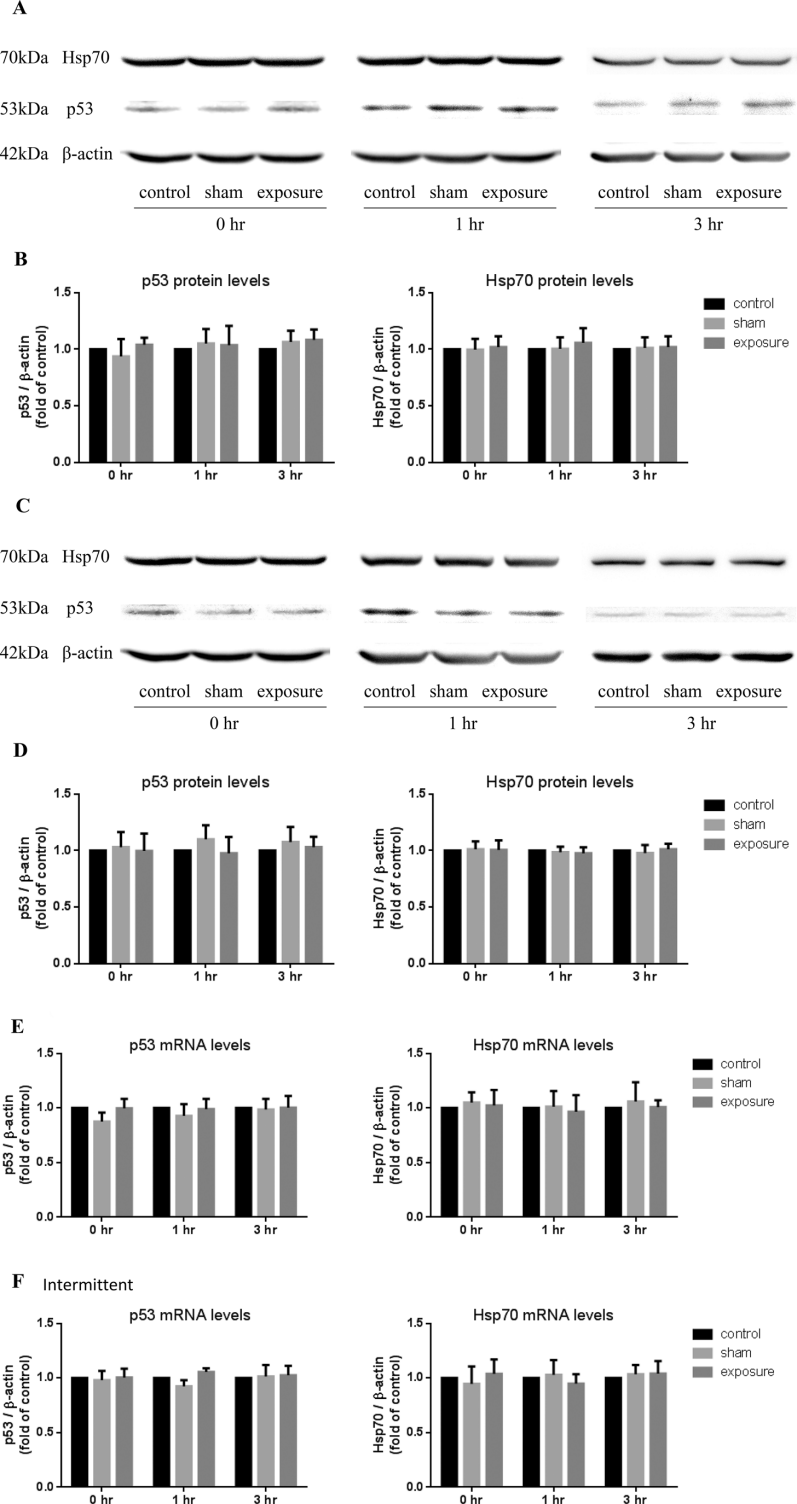
**Effects of exposure to 50 Hz MF on p53 and Hsp70 expression in AC16 cells.** Western blot analysis of p53 and Hsp70 in AC16 cells at 0, 1 and 3 h after 50 Hz MF exposure for 1 h continuously (A) or 75 min intermittently (C). The protein expression of β-actin was used as loading control. The intensity of p53 and Hsp70 blots were quantified in both the continuous (B) and intermittent (D) exposure modes. The data were normalized over the corresponding controls (=1), presented as the mean±s.e.m. from three independent experiments, each repeated three times. Gene expression of p53 and Hsp70 in mRNA levels were detected by quantitative real-time PCR at 0, 1 and 3 h after 50 Hz MF exposure for 1 h continuously (E) or 75 min intermittently (F). The mRNA expression of β-actin was used as loading control. The data were normalized over the corresponding controls (=1), presented as the mean±s.e.m. from three independent experiments.

**Fig. 6.**
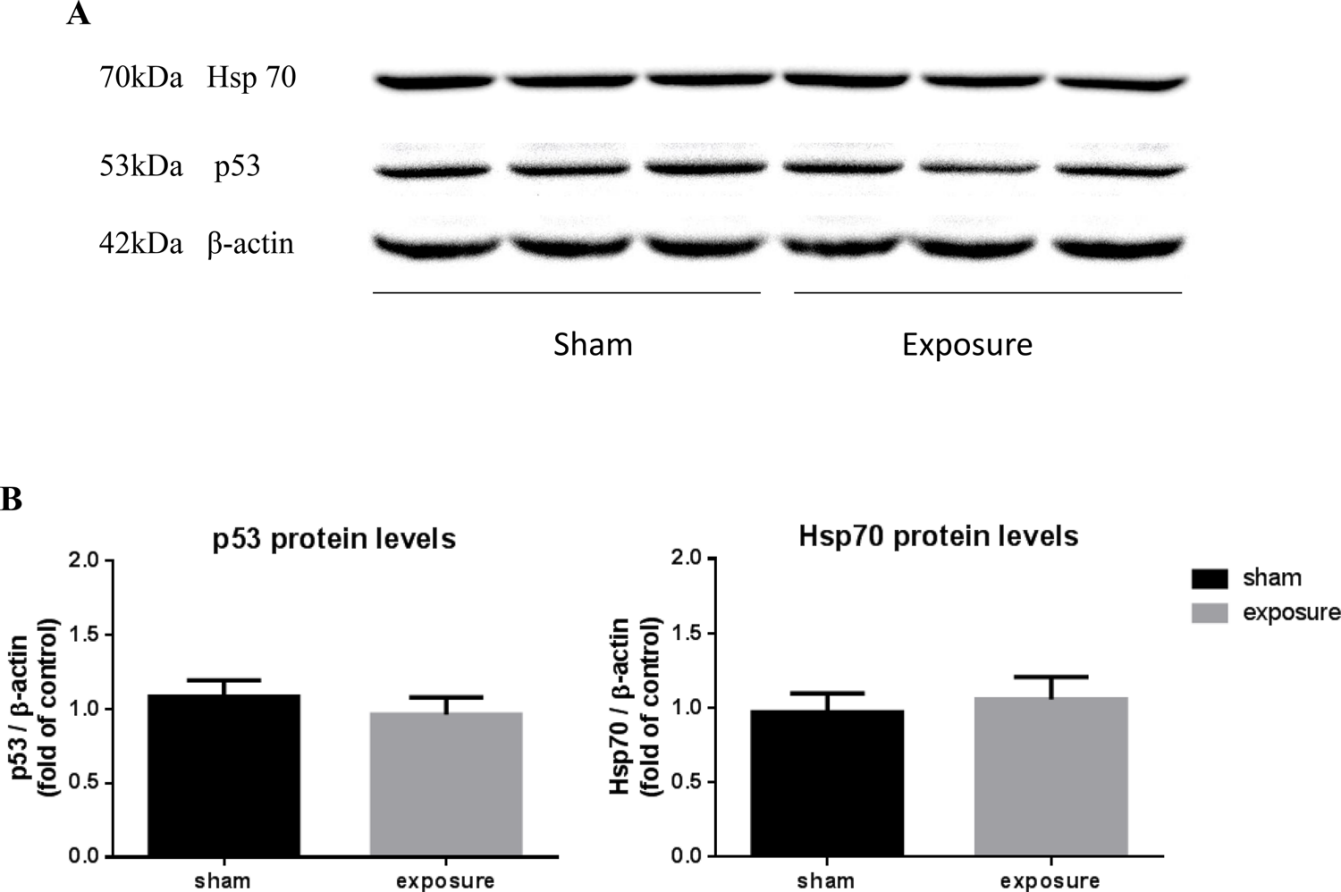
**Effects of short-term exposure to 50 Hz MF on p53 and Hsp70 protein levels in rat hearts.** (A) Representative blots for p53 and Hsp70 after sham- or MF-exposure, using β-actin as loading control. (B) Quantification of p53 and Hsp70 were shown as relative protein expression after normalization to β-actin. The representative blots were replicates from different animal samples, and each sample was performed three times. The data represent the mean±s.e.m. (*n*=10 for each group).

Finally, we investigated the gene expression and protein level of p53 and Hsp70, which responded to DNA damage rapidly. MF exposures were carried out in continuous (Fig. 5A,B,E) or intermittent (Fig. 5C,D,F) modes in cardiomyocytes. Measurements were performed immediately after the treatment (0 h) or after 1 and 3 h of post-stress recovery. Both mRNA and protein levels of p53 and Hsp70 were not altered at these three time points in the MF-exposed group compared to the sham group (Fig. 5A–F). Additionally, in the heart tissue of exposed rats, the protein levels of p53 and Hsp70 were not changed (Fig. 6A,B).

## DISCUSSION

The current study showed that neither continuous nor intermittent exposure to a 50 Hz MF at 100 µT induced DNA damage in cardiomyocytes, which were evaluated by primary DNA damage, cell cycle distribution, intracellular ROS level, changes in the redox status and expression levels of p53 and Hsp70.

Endogenous factors, including chemicals, ionization and non-ionizing radiation, can lead to DNA damage. Of the two types of DNA damage, although double-strand breaks are usually more critical and often lethal for cells, single-strand breaks occur from 5- to 2000-fold more than double-strand breaks (Bradley and Kohn, 1979). Therefore, the alkaline comet assay was used in our study to detect the single-strand breaks of DNA. We found that the DNA migration parameters of AC16 cells exposed to 50 Hz MF were not significantly changed, indicating that single-strand breaks of DNA in cardiomyocytes were not affected by ELF-MF exposures.

In order to further investigate whether ELF-MF increased DNA damage in AC16 cells, we detected the cell cycle distribution and p53 of AC16 cells after MF exposure. The activation of p53 in response to DNA damage could induce arrest within the G1 or G2 phase of the cell cycle (Levine, 1997; Hermeking et al., 1997). This reaction was so sensitive that as little as one DNA double-strand break could initiate p53-invoked cell cycle arrest. In our study, cell cycle distribution of AC16 cells was detected at 0, 4 and 16 h after MF exposure, and no significant difference was observed compared with the sham and the control groups. Our *in vivo* study also did not observe any increase of p53 protein levels in rat heart. These results indicated that ELF-MF exposure did not induce genomic instability via DNA damage and cell cycle arrest in cardiomyocytes.

At 50 Hz MFs, the photon energy is about 10^–12^ of the energy required to break the weakest chemical bond (Valberg et al., 1997). Therefore, some studies that observed DNA damage after ELF-MF exposure claimed that this effect was produced by indirect or secondary biochemical changes in cells. Free radicals mediated by the Fenton reaction were considered the most likely reason. Martinez-Samano et al. reported that decreased GSH in heart after 2 h of 60 Hz, 2.4 mT MF exposure might be induced by the altered metabolism of free radicals (Martínez-Sámano et al., 2010). In contrast, our study showed no increase in total intracellular ROS level of AC16 cells after exposure to a 50 Hz, 100 µT MF, while GSH was not changed. These inconsistent results may be due to different experimental conditions, especially the magnetic flux gap and exposure time. However, the average exposure to ELF-MFs of the general public in European countries is between 0.01 and 0.1 µT, and scarcely any exposure exceeds 100 µT at 50 Hz (Gajšek et al., 2016). The experimental conditions in our study were closer to the real environment.

Hsp70, as a stress-response protein, can be induced by many different external factors, such as temperature, various chemicals, oxidative stress, heavy metals, ionizing and non-ionizing radiation. Whether ELF-MFs are able to induce hsp70 expression still remains a controversial issue. For example, hsp70 has been found to increase after ELF-MF exposure (Tokalov and Gutzeit, 2004; George et al., 2008; Blank and Goodman, 2009), while others reported negative results (Mariucci et al., 2010; Villarini et al., 2017, 2013). Because the experimental conditions were different from each other in regard to equipment used to generate MFs, exposure protocol (frequency, flux intensity and/or time) and biological material (cell lines and animal species, strain and age), the results of various studies may be different.

In conclusion, the present study demonstrated that 50 Hz MF exposure at 100 µT did not induce detectable primary damage on DNA in cardiomyocytes. However, further studies are still needed to better understand the effects of MF exposure on the heart.

## MATERIALS AND METHODS

### Animals

All of the animals used in the study were housed in the Animal Centre of Tongji Hospital, Tongji Medical College, Huazhong University of Science and Technology (HUST, Wuhan, China). The experimental protocols were approved by the Animal Care and Use Committee of HUST, and were performed according to the Principles of Laboratory Animal Care, the Office for Protection from Research Risks (OPRR) Public Health Service Policy on Humane Care and Use of Laboratory Animals (revised 2015), and the U.S. Animal Welfare Act 1966 (as amended in 2013) were followed, as well as the specific national laws (i.e. the current version of the Japanese Animal Welfare Law 2013) (Zhou et al., 2016).

Twenty male Sprague-Dawley (SD) rats (8 weeks of age) were housed in standard laboratory cages in a temperature-controlled room (22±2°C) with light/dark alternating for 12 h, starting light at 07:00 h. The rats were randomly divided into two groups: exposure group (*n*=10) and sham-exposure group (*n*=10). The exposure group was exposed to ELF-MF for 15 h a day, from 17:00–08:00 h, for 7 days continuously. As a control, the sham-exposure group was placed in the unpowered exposure system at the same time. All the animals were euthanized the day after the last exposure to avoid acute effects due to handling, stress and so on. Saline was perfused to minimize blood contamination in cardiac tissue. Then, cardiac tissue samples were flash frozen in liquid nitrogen for western blotting. All animals were housed in polycarbonate ventilated cages (45 cm×20 cm×35 cm, L×W×H) with polycarbonate supporting floors, and received irradiated corncob bedding. The cages were changed every 2 weeks, and the bedding was changed weekly. The food and drinking water were sterilized using filtration and ozone.

### Exposure system

The exposure system has been reported previously (Zhou et al., 2016; Lai et al., 2016). Briefly, the exposure system, consisting of a pair of parallel coils (200 cm×70 cm×200 cm, L×W×H), was built by Yite Electric (Wuhan, China). Horizontal bronze wires were used to conduct the current and generate a vertical MF at 50 Hz and 100 µT (Misakian et al., 1993). In order to generate the uniform MF, the wiring ratio of the five coils was 2:1:1:1:2, and the 3% uniformity area for each floor was 150 cm×50 cm (L×W). The wires were fixed tightly to the framework in order to decrease vibration and noise. The distributions of the 100 μT ELF-MFs are shown in Fig. S1. Meanwhile, a sham-exposure system (unpowered exposure system), exactly the same as the exposure system but without power, was located in separated and non-adjacent rooms which had the same room conditions, and all breeding conditions were as the exposure system.

### Cell culture and treatments

The human ventricular cardiomyocyte cell line AC16 cells were purchased from American Type Culture Collection (ATCC). AC16 cells were routinely grown in Dulbecco's modified Eagle's medium (DMEM, Gibco, Shanghai, China) containing 10% fetal bovine serum (FBS, Gibco, Shanghai, China) and 2% L-glutamine (Sigma-Aldrich, Shanghai, China) in an atmosphere of 95% air and 5% CO_2_ at 37°C. Cells were maintained in 25 cm^2^ plastic flasks and sub-cultured every 2 days at 1:4 ratio in 60-mm dishes or six-well plates with a cover slip. After 24–48 h cell seeding, cells were exposed to a 50 Hz, 100 µT MF continuously for 1 h or intermittently (15 min field on/15 min field off) for 75 min at 22°C. Negative control (cells stayed in incubator) and sham-exposed cells (without MF exposure at 22°C) were set up simultaneously. Experiments were repeated three times.

### Alkaline comet assay

The comet assay was carried out according to the method reported by McNamee et al. with little modification (McNamee et al., 2000), and the sensitivity is shown in Fig. S2. After exposure, AC16 cells were washed with PBS, incubated in 0.25% trypsin with 0.02% EDTA for 1 min at 37°C, then stopped by DMEM with 10% FBS, and centrifuged at 500 ***g*** for 5 min. Cells were washed with ice-cold PBS, before being re-suspended in ice-cold PBS to 5×10^5^ cells/ml. The control cells with sham exposure were divided into two equal parts, one of them was placed for 5 min in ice-cold 2 µM H_2_O_2_ prepared in PBS as positive control. A 40 µl aliquot of cell suspension from each tube was mixed with 400 µl pre-thawed 0.5% low-melting-point agarose at 37°C. A 100 µl aliquot of cell-agarose suspension was immediately pipetted onto 1% normal-melting-point agarose pre-coated slides, spread using a cover glass, and placed in a capsule on ice for about 10 min to solidify. Removing the cover glass, the slides were immersed in a freshly prepared cold lysis buffer (2.5 M NaCl, 100 mM Na_2_EDTA, 10 mM Tris base, 1% N-laurylsarcosine, 1% Triton X-100, 10% DMSO, pH 10.0) and maintained for 30 min on ice. The slides were rinsed with ion-free water, then put into solution (2.5 M NaCl, 100 mM Na_2_EDTA, 10 mM Tris base, pH 10.0) with 1 mg/ml proteinase-K supplemented immediately before use. After incubation for 1 h at 37°C, the slides were rinsed again and placed in a horizontal gel-electrophoresis tank filled with fresh ice-cold electrophoresis buffer consisting of 1 mM Na_2_EDTA, 200 mM NaOH, pH 13.0 for 20 min on ice to unwind DNA, then electrophoresed at constant voltage (1.5 V/cm, 300 mA) for 30 min on ice. In order to avoid the potential position effect during electrophoresis, two parallel replicates were prepared for each sample. The positions of each replicate were switched during different electrophoresis processes. Following electrophoresis, the slides were drained, rinsed twice with ion-free water and then placed in 70% ethanol for 10 min, before air-drying in 37°C for 30 min. Subsequently, the agaroses were stained with propidium iodide (PI; 50 µg/ml, Sigma-Aldrich) and RNAse A (50 µg/ml, Invitrogen) for 1 h. All steps were conducted in the dark to prevent additional DNA damage. Comet images were obtained using a NIKON fluorescence microscope and analyzed by CASPLab program. By dragging the measurement frame onto a cell, the parameters, including head radius, tail length, tail DNA percentage, tail moment and comet length were analyzed. At least 50 cells were randomly selected from each of the two replicates (Końca et al., 2003). Since the distribution of comets was non-normal, the mean values of the medians of the replica were used for comparison (Moller and Loft, 2014).

### Cell cycle analysis

After exposure, the AC16 cells were cultured for an additional 0, 6 or 16 h, then trypsinized and collected. Samples were centrifuged at 500 ***g*** for 5 min, rinsed twice with ice cold PBS, and fixed in 70% ethanol overnight at 4°C. The cells were rinsed twice again, then stained with 50 µg/ml PI containing 50 µg/ml RNase A and 1% Triton X-100 for 1 h, before being analyzed by flow cytometry. As a positive control, cells were treated with 1 μM of nocodazole (cat. no. HY-13520, MedChemExpress, Shanghai, China) corresponding to MF exposure to inhibit cell cycle progression. Nocodazole was removed by repeated washes with DMEM after treatment, and cultures returned to incubator.

### Determination of ROS

ROS levels were detected with the ROS assay kit (Beyotime Biotechnology, Shanghai, China) according to the manufacturer's instructions. After exposure, cells were harvested, washed with PBS and incubated with serum-free medium containing 10 μM 2′,7′-dichlorodihydrofluorescein diacetate (DCFH-DA) in the dark at 37°C for 30 min. Then the samples were rinsed with PBS and analyzed for the fluorescence of 2′,7′-dichlorodihydrofluorescein (DCF) by flow cytometry.

### Reduced and oxidized glutathione assay

The ratio of reduced/total GSH was measured using the GSH and GSSG Assay Kit (Beyotime Biotechnology, Shanghai, China) according to the manufacturer's instructions. Briefly, after treatment, cells were washed with ice-cold PBS and harvested by scraping into 5% metaphosphoric acid (MPA), vortexed thoroughly, then flash-frozen in liquid nitrogen and thawed at 37°C in a water bath repeatedly. The samples were then placed on ice for 5 min and centrifuged at 10,000 ***g*** for 10 min at 4°C to collect the supernatants for detection. To determine the total glutathione, the supernatants were diluted and mixed with detection-working solution in a 96-well plate, incubating at 25°C for 5 min. Finally, NADPH was added to each well, before the plate was read by a synergy 2 microplate reader (Bio-tech, USA) at 412 nm for every 5 min. To determine the GSSG (oxidized glutathione), the diluted supernatants were firstly mixed with GSH scavenging solution and incubated at 25°C for 60 min. Then the samples were mixed with detection working solution, added with NADPH, and read by microplate reader. The GSSG standards were used as the standard curve and treated corresponding to the samples. The value of reduced GSH was obtained from the total GSH minus the GSSG.

### RNA extraction, cDNA synthesis and real-time PCR

Total RNA was extracted from harvested cells using TRIzol (TransGen Biotech, Beijing, China), according to the manufacturer's instructions. The cDNAs were obtained by reverse transcription-PCR using PrimeScript™ RT reagent Kit with gDNA Eraser (Takara, Dalian, China). Real-time PCR was performed using TB Green Premix Ex Taq II (Takara, Dalian, China) on the 7900 Real-Time PCR System (Applied Biosystems, USA). The reaction comprised of an initial pre-denaturation step at 95°C for 1 min, followed by 40 cycles at 95°C for 5 s, 60°C for 34 s, and a final dissociation stage at 95°C for 15 s, 60°C for 1 min, 95°C for 15 s. The primers were designed on the basis of the published sequence of p53, Hsp70 and β-actin (house-keeping gene). The following primers were used: p53, 5′-GTACCACCATCCACTACAACTACAT-3′ (forward) and 5′-AAACACGCACCTCAAAGCTG-3′ (reverse); Hsp70, 5′-GATGTGTCGGTTCTCTCCATTG-3′ (forward) and 5′-CTTCCATGAAGTGGTTCACGA-3′ (reverse); β-actin, 5′-TGGATCAGCAAGCAGGAGTATG-3′ (forward), 5′-GCATTTGCGGTGGACGAT-3′ (reverse). Each sample was analyzed in triplicate.

### Western blot analysis

Inducible Hsp70 protein and p53 protein levels were determined by western blot analysis using respective specific antibodies (cat. no. ab181606 and cat. no. ab1101, Abcam, Shanghai, China). The cell pellets were re-suspended in ice-cold cell lysis buffer with proteases inhibitors (Boster, AR1182, Wuhan, China), and rotated for 20 min at 4°C. The cardiac tissues were homogenized on ice. All homogenates were centrifuged at 12,000 ***g*** for 5 min at 4°C, supernatants were collected and the protein concentration was measured by BCA Protein Assay Kit (Beyotime Biotechnology). Equal amount proteins (20 µg) were separated by 10% SDS-polyacrylamide gels before being transferred to polyvinylidene fluoride membranes. The membrane was blocked with Tris-buffered saline-Tween 20 (TBS-T), pH 7.5, containing 5% BSA for 1 h at room temperature and incubated at 4°C overnight with specific antibodies to Hsp70, p53 and β-actin in 5% blocking solution. After three washes of TBS-T, for 5 min each, the membrane was incubated for 1 h in HRP-conjugated antibody at a dilution of 1:5000, and washed three times with TBS-T. The enhanced chemiluminescence system was used to visualize the bands. The images were captured with the Luminescent Imaging System (Tanon, Shanghai, China) and inverted to a bright background. The blots were quantified by densitometry with Gel Pro analysis software. The obtained values were corrected by corresponding β-actin (cat. no. ab8226, Abcam). Each experiment was repeated three times.

### Statistical analysis

Data were presented as mean±s.e.m. and differences between two groups were determined by pairwise comparison using an unpaired Student’s *t*-test. Comparisons among multiple groups were performed by one-way ANOVA with post hoc analysis using Fisher’s least-significant difference (LSD). When data did not conform to normal distribution, the Mann–Whitney *U*-test was applied to perform pair-wise comparisons, and the nonparametric Kruskal–Wallis test was applied for the multiple groups. *P*<0.05 was considered statistically significant.

## Supplementary Material

Supplementary information
