# Supplementary material for: Exposure to a 50 Hz magnetic field at 100 µT exerts no DNA damage in cardiomyocytes
Source: Biol Open. 2019 Aug 7;8(8):bio041293. doi: 10.1242/bio.041293 (PMC6737969; doi:10.1242/bio.041293)
Supplement: Supplementary information [file biolopen-8-041293-s1.pdf]

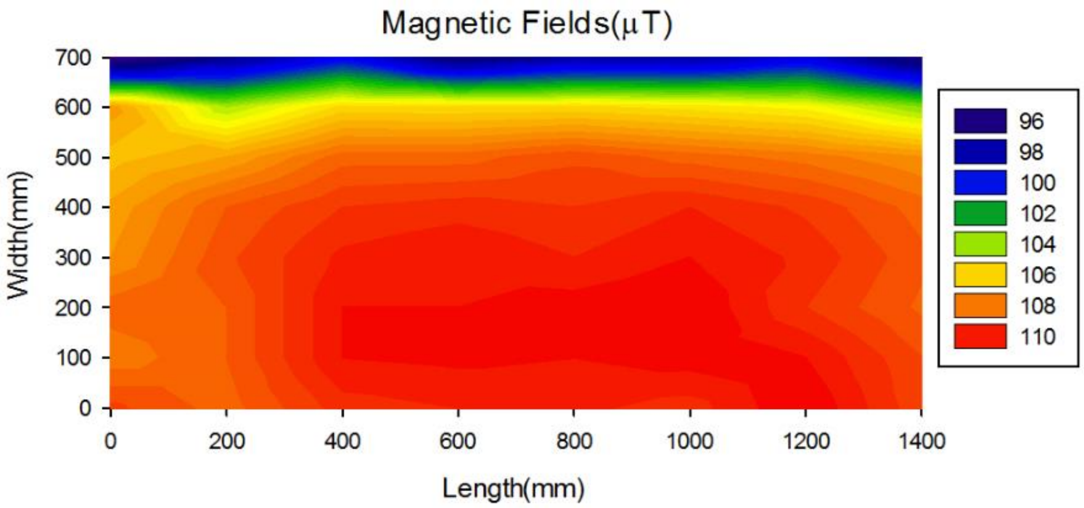

Figure S1. The distributions of the 100μT ELF-MFs was uniform.

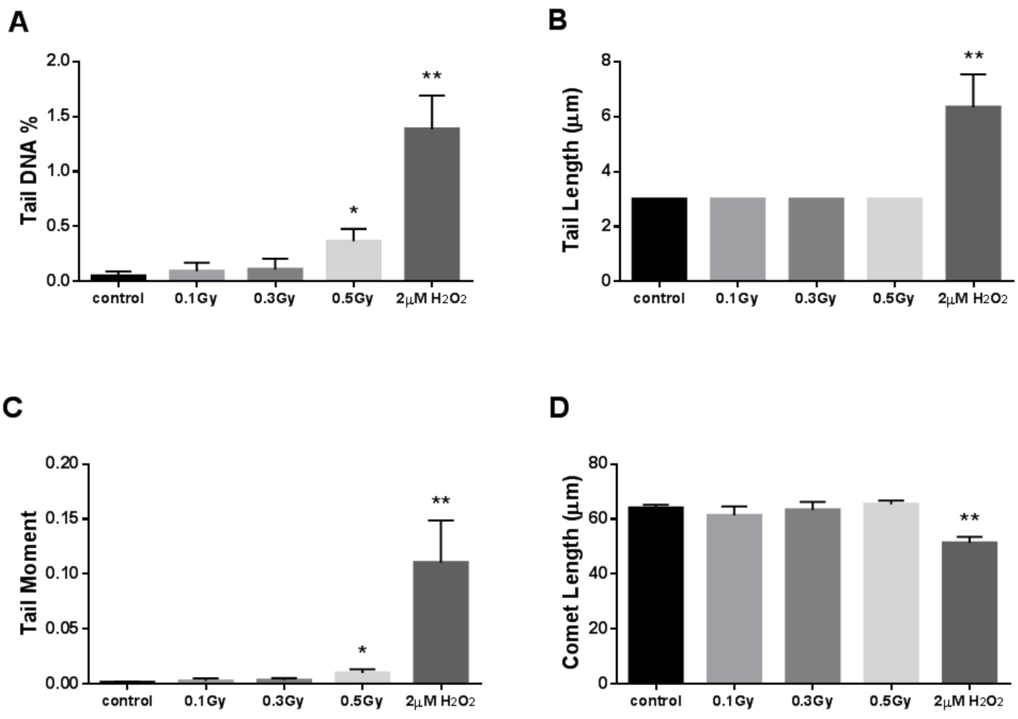

Figure S2. DNA damage in AC16 cells exposed to X-irradiation. Levels of DNA damages were evaluated by the alkaline comet assay, expressed as tail DNA% (A), tail length (B), tail moment (C) and comet length (D). The negative control (control) represented unstressed cells kept under normal culture conditions. The positive control used negative control cells exposed in ice-cold 2µM hydrogen peroxide for 5 min. Each bar shows the mean±SEM of three independent experiments. A minimum of 50 cells were collected from each replica of a sample, using the mean values of the medians of the two replicas for comparison (**$P < 0.01$, *$P < 0.05$ vs. control).

Biology Open • Supplementary information
